# Primary Conjunctival Rhabdomyosarcoma in a Pediatric Patient

**DOI:** 10.7759/cureus.6310

**Published:** 2019-12-06

**Authors:** Laura Morales R, Angelina Álvarez, José Esguerra, Maria Camila Prada Avella, Fernando Rojas

**Affiliations:** 1 Radiation Oncology, Instituto Nacional de Cancerologia, Bogota, COL; 2 Radiation Oncology, Instituto Nacional de Cancerología, Bogota, COL; 3 Pediatric Oncology, Instituto Nacional de Cancerologia, Bogota, COL; 4 Oncological Ophthalmology, Instituto Nacional de Cancerologia, Bogota, COL

**Keywords:** rhabdomyosarcoma, conjunctival tumor, pediatric cancer, orbital tumor

## Abstract

Rhabdomyosarcomas are neoplasms with a high degree of malignancy and arise from the embryonic mesenchyme. They represent approximately 5% of all pediatric tumors and their main locations are the head and neck (45%), trunk (40%), and extremities (15%). Twenty-five percent to 30% of the head and neck rhabdomyosarcomas appear in the orbit; however, its origin from the conjunctiva is rare, with few case reports published in the literature.

We present the case of a five-year-old girl with a diagnosis of primary embryonic rhabdomyosarcoma of the conjunctiva, treated with surgery and chemotherapy. After completing the treatment, it was followed up with controls for oncological ophthalmology, pediatric hematology-oncology, and radiotherapy oncology every six months with magnetic resonance of the orbits. Two years after the end of treatment, the patient is disease-free.

Conjunctiva rhabdomyosarcoma is a rare lesion, with few previously reported cases. In the reported case, the histopathological findings and positivity of the different immunohistochemical markers allowed a definitive diagnosis of rhabdomyosarcoma. The excellent prognosis of this pathology is probably linked to the early diagnosis of the disease and the timely administration of radical treatment.

It is essential to be able to identify conjunctival rhabdomyosarcoma from its clinical and histopathological characteristics in order to achieve early diagnosis and provide adequate treatment to patients.

## Introduction

Rhabdomyosarcomas are neoplasms with a high degree of malignancy, which arise from the embryonic mesenchyme and have the potential to differentiate into striated muscle. They can appear almost anywhere on the body, including the orbit and eyeball, corresponding to 9% of all these tumors [1]. About 70% of cases occur before 10 years, with a peak incidence between two and five years; in tumors of the orbit, this peak usually occurs at around six years old [2]

Although they are the most frequent primary tumors of the orbit in pediatric patients and the botryoid variant can compromise the conjunctiva in 5% to 8% of cases, primary conjunctival rhabdomyosarcomas are very rare, with few cases reported in the literature [3-4]. In Shields’ case series, conjunctival rhabdomyosarcomas were 12% (four patients) of all ophthalmic rhabdomyosarcomas [5].

As for their clinical manifestations, these tumors may present as a conjunctival mass or as a hyperemic area [4]. Some of its differential diagnoses are conjunctival lymphomas, lipomas, myxomas, intraepithelial conjunctival neoplasms, and other benign entities such as conjunctivitis or granulomas [6].

Generally, rhabdomyosarcomas are classified into four morphological categories: embryonic, alveolar, botryoid, and pleomorphic. The botryoid subtype is also considered as a variant of the embryonic type, which is characterized by a papillary configuration [4,7].

During the twentieth century, the only treatment was radical surgery; however, due to the development of new treatment strategies, such as chemotherapy and radiotherapy, supported by the creation of groups such as the International Rhabdomyosarcoma Study Group (IRSG), overall survival has increased from 15% or 25% to more than 70% in recent decades [8]. As for orbital rhabdomyosarcoma, it presents an excellent prognosis with 10-year overall survival rates of up to 87% [4,7,9].

Nowadays, most protocols for the management of rhabdomyosarcomas are based on risk stratification, which is determined by multiple staging systems, histology, and tumor location [10]. Among the staging systems, we find the International Society for Pediatric Oncology (SIOP) system where a pretreatment staging is performed based on tumor size, lymph node involvement, and the presence of metastases (TNM); and the staging was proposed by the IRS group based on the level of tumor resection [11-12].

## Case presentation

**Figure 1:**
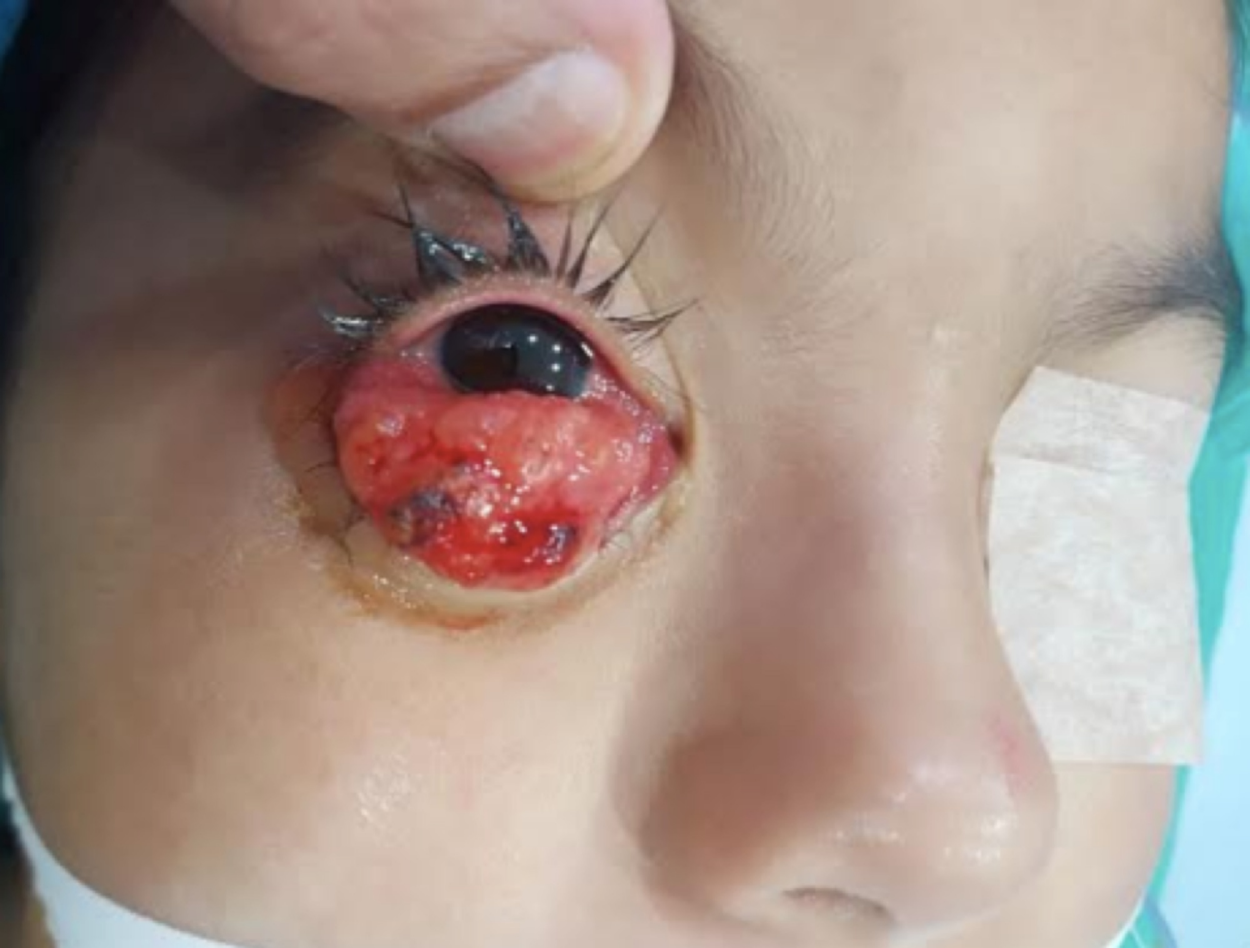
Right conjunctival mass

**Figure 2:**
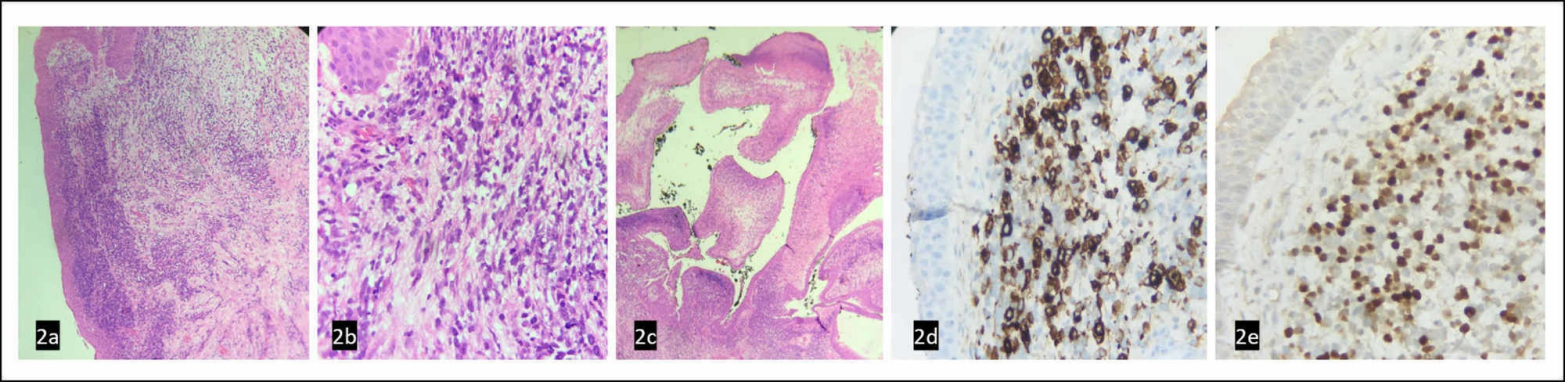
Immunohistochemical stains (2a) Hematoxylin eosin 4X: Squamous mucosa with undifferentiated malignant tumor; (2b) Hematoxylin eosin 40X: Atypical cells in myxoid background; (2c) Typical polypoid appearance of botryoid variant; (2d) Fi Hematoxylin eosin 10X: Desmin; (2e) Hematoxylin eosin 10X: Myogenin

A five-year-old patient, with a clinical picture of 4 months of evolution, consisting of the appearance of the right conjunctival mass of progressive growth. Initially, she was evaluated extra-institutionally, where she had a partial mass resection. On that occasion, the pathology reported an embryonic rhabdomyosarcoma, the reason why she was referred to our institution. The patient was then evaluated by the oncology ophthalmology service, which, on ophthalmological examination, observed a mass that occupied the entire extent of the lower conjunctiva, causing palpebral ectropion of friable appearance, with soft and papillomatous edges (Figure 1). She was taken to an esophageal biopsy of the conjunctival mass. In the pathology, a squamous mucosa massively compromised by a malignant tumor of small, round, and blue cells was observed, with atypia, mitosis, fusocellular cells, and myxoid areas with the subepithelial layer phenomenon, creating a polypoid appearance. The immunohistochemical study revealed the positivity of the cells for desmin and myogenin and negativity for the common leukocyte antigen (LCA), WT-1, PAX5, TdT, CD99, and fli 1 (Figure 2), thus confirming the diagnosis of embryonic rhabdomyosarcoma.

Subsequently, it is staged as a botryoid variant embryonal rhabdomyosarcoma of the right conjunctiva (favorable location), low-risk subgroup Ia, T1 (<5 cm), N0 (no ganglionic involvement), M0 (no metastasis), post-surgical clinical group (IRS), IIIa (localized size or with local extension, with macroscopic residual disease, only biopsied). The patient received chemotherapy for low-risk sub-group Ia rhabdomyosarcoma. Treatment was provided with eight cycles of VAC regimen chemotherapy (vincristine, adriamycin, and cyclophosphamide). At the end of this treatment, a contrasted magnetic resonance of orbits was requested, where no residual mass was evidenced. Taking into account that the patient had not been taken to an oncological resection, but, instead, the surgical intervention performed was an excisional biopsy, the multidisciplinary group of treating physicians proposed to provide treatment with external radiotherapy after chemotherapy. The case was presented at a radiation oncology meeting and, taking into account the possible side effects of radiotherapy, it was decided to provide expectant treatment, and so the patient was left on follow-up with imaging controls every six months.

Three years after the end of treatment, the patient remains clinically and imaging free of disease, with an adequate visual acuity and without symptoms suggestive of toxicity secondary to treatment.

## Discussion

Rhabdomyosarcoma is the most common neoplasm of the orbit within the pediatric population, representing approximately 5% of all pediatric tumors [3]. The main location of this tumor is the head and neck, and 25% to 30% of the rhabdomyosarcomas of tumors originating in this anatomical area appear in the orbit; nevertheless, its origin from the conjunctiva is exceptional with less than 20 cases reported in the literature [2-4].

In this case, the clinical presentation of the tumor was the usual one, in accordance with what has been reported by other authors [13]. Despite this, the initial diagnostic suspicion was not that of a rhabdomyosarcoma, given the rarity of the location. However, the pathology and various immunohistochemical markers allowed for diagnosis to be established at a convenient time, so that appropriate treatment could be offered while preserving the organ.

The tumor was staged as an Ia group according to the IRSG, and the current recommendation for this group of tumors is to perform a surgical resection and subsequently provide adjuvant treatment with VAC regimen chemotherapy. However, it was contemplated to provide treatment with radiotherapy since the patient was not taken to oncological resection but to excisional biopsy. Taking into account the limited available literature on conjunctiva rhabdomyosarcoma and its treatment and that radiotherapy could lead to late toxicity that is not negligible, it was decided to leave the patient under observation. Also, the results of the clinical trial of the International Society of Pediatric Oncology Malignant Mesenchymal Tumor (MMT-89) suggested that the omission of radiotherapy was justifiable for some patients if they were treated according to the guidelines of the IRSG [14]. Similarly, Brichard et al. reported the case of a primary conjunctival rhabdomyosarcoma treated exclusively with chemotherapy with promising results [15].

Our case emphasizes the importance of a careful histopathological review of all conjunctival masses in children in order to achieve timely diagnosis and treatment.

So far, the patient is free from relapse. The favorable prognosis may be related to tumor location, early detection, and embryonic histological type. Taking into account the excellent prognosis, the goals of treatment are, in addition to curing the disease, minimizing late sequelae secondary to treatment.

## Conclusions

It is necessary to know that in the pediatric population, rhabdomyosarcoma can originate from the conjunctiva. Knowledge of its clinical, histopathological, and imaging characteristics is essential in order to achieve early diagnosis. It is essential to propose a multidisciplinary treatment where the risks and benefits of the different therapeutic tools are always evaluated.
